# AI-assisted grading and personalized feedback in large political science classes: Results from randomized controlled trials

**DOI:** 10.1371/journal.pone.0328041

**Published:** 2025-08-19

**Authors:** Tobias Heinrich, Spencer Baily, Kuan-Wu Chen, Jack DeOliveira, Sanghoon Park, Navida Chun-Han Wang

**Affiliations:** 1 Department of Political Science, The University of Houston, Houston, Texas, United States of America; 2 Department of Political Science, University of South Carolina, Columbia, South Carolina, United States of America; 3 Institute of Political Science, Academia Sinica, Taipei, Taiwan; 4 Kangwon Institute of Unification Studies, Kangwon National University, Chuncheon, Gangwon, Republic of Korea; 5 Department of Political Science, University of Michigan, Ann Arbor, Michigan, United States of America; Nanyang Technological University, SINGAPORE

## Abstract

Grading and providing personalized feedback on short-answer questions is time consuming. Professional incentives often push instructors to rely on multiple-choice assessments instead, reducing opportunities for students to develop critical thinking skills. Using large-language-model (LLM) assistance, we augment the productivity of instructors grading short-answer questions in large classes. Through a randomized controlled trial across four undergraduate courses and almost 300 students in 2023/2024, we assess the effectiveness of AI-assisted grading and feedback in comparison to human grading. Our results demonstrate that AI-assisted grading can mimic what an instructor would do in a small class.

## Introduction

Cultivating critical thinking is a core goal of political science education [21,32]. Traditionally, instructors use essays or short-answer questions (SAQs) where students synthesize complex ideas and receive personalized feedback from the instructor [3,13,26]. In large classes, however, this practice demands significant time and focus from instructors, conflicting with research and service duties. Consequently, large classes are likely to expose students to fewer SAQs and more multiple choice questions (MCQs) [18], potentially compromising student learning.

Our study directly addresses this challenge by testing whether large language models (LLMs) can increase instructors’ productivity grading SAQs to provide the personalized feedback that instructors typically reserve for smaller classes. We focus on political science courses, but our results hold broader implications for any large-enrollment setting seeking to balance rigorous assessment with instructors’ time constraints. Throughout, we use “grading” and “grades" to mean both scoring and giving detailed, personalized feedback. When a distinction is necessary, we will make it clear.

We propose leveraging this capability by providing LLMs with complex, instructor guided prompt templates to grade a new answer. The prompt includes the text of the SAQ, the instructor’s criteria for a good answer, multiple examples of student responses paired with the instructor’s grades and personalized feedback, and a new, ungraded student answer. Based on this input, the LLM predicts the next sequence of words, namely, the grade [4,28]. This approach, known as in-context or few-shot learning, encourages the model to align its output with the instructor’s standards. When effective, the model produces grades and feedback that are consistent with those of the instructor. This approach holds significant potential for boosting instructional productivity, as it can be scaled to accommodate large numbers of students and many SAQs.

To test whether this approach succeeds at mimicking an instructor’s personalized grading, we analyze data from a randomized controlled trial (RCT) conducted in four political science undergraduate classes during the 2023/2024 academic year. We randomly assigned ten students’ responses for each SAQ to either intensive human or AI-based grading. We evaluate the approach’s numerical grade discrimination (how a student’s general ability answering questions related to the test predicts the particular SAQ’s score), the probability of students asking for a human regrade (as a measure of students’ perception of adverse grading errors), and students’ own evaluation of the helpfulness of the written feedback.

Our data covers 271 students taking 26 tests and producing 3,080 SAQ answers, 88% of which were graded with AI assistance. The empirical results suggest that our prompts can effectively make LLMs mimic key aspects of the instructor’s grading. Some differences do occur which tend to be minor and not systematic across classes. In short, LLMs manage to augment the instructor’s grading productivity so that highly personalized grading, a hallmark of smaller classes, can be carried out in larger classes without prohibitive professional opportunity costs.

This study demonstrates the potential of LLMs to mimic instructor grading, but it does not directly assess student learning outcomes and critical thinking skills. Our notion of high-quality and detailed feedback rests on the idea that grading a smaller set of answers with more attention leads to better feedback, and if done more often, helps students develop their critical thinking skills. However, understanding if AI feedback improves learning requires long-term studies tracking student performance over time and across classes. We would need students who never received AI feedback, which was the case for nobody in our study. Thus, answering this question is beyond the design of the study.

Further, our design also does not allow for a comparison of our results to a large class with many SAQs, all of which instructors personally grade with great care and attention. This is a “phantom counterfactual” [30], as associated professional opportunity costs from doing so are so steep that instructors would not set up their classes that way.

Our analysis shows evidence for an increase in instructors’ productivity in grading SAQs. Our approach let us deliver feedback and grades to an average class size of almost 70 students that instructors may only provide if there were only a fourth or a third of the students, mirroring productivity gains when co-working with AI on tasks that have been reported in studies [5,7,17,19,24].

Our design used anticipated productivity gains to enable more students to receive detailed feedback on a greater number of SAQs. However, alternative allocations are possible [1]. For example, some instructors may use freed-up time to increase research output for professional gain. Others, or their teaching assistants, might hold more frequent and longer 1-on-1 meetings with students. And perhaps some might increase leisure time. Which activities instructors, administrators, and other higher education stakeholders prioritize from the productivity gains is an important ongoing conversation [6,16,20,25,32]. However, lessons for this conversation are beyond the scope of this paper, which examines the feasibility of increasing grading and feedback productivity.

## Research design

Our approach to grading SAQs, which was posted on the Open Science Foundation website on January 13, 2024, available at https://osf.io/hpc7g, followed similar steps in each of the four classes. Deviations from the pre-analysis plan are summarized in S1 Appendix. Ethical approval was given by the Institutional Review Board of the University of South Carolina for one class in Fall 2023 on September 7, 2023 (Pro00131778) and for the others in Spring 2024 on January 16, 2024 (Pro00134602).

At the start of the semester, each instructor explained our AI-assisted grading approach to students, including the rationale behind it, which matched this paper’s arguments. While the approach was implemented as part of the course policy, students could choose whether to allow their data to be used for research in exchange for minor extra credit. Informed consent could be given and withdrawn at any time until the last day of class, and 99% of enrolled students ultimately consented. The consent form is provided in S2 Appendix. Instructors remained unaware of students’ choices throughout the semester.

Each bigger test, midterm, final, and smaller quiz, which we will call “test" going forward, was conducted via the university’s learning platform and included a mix of SAQs and MCQs. For the grading of each SAQ, the instructor developed an LLM prompt template into which the ungraded answers were iteratively inserted for grading. In S4 Appendix, we present one synthetic illustration of the entire process, including the prompt template, using an SAQ on an article by Kim and Pelc on the domestic politics of U.S. trade [11] from one class as an example.

The prompt template for grading SAQs contains five parts:

1. **Preamble** – brief instructions telling the LLM that its task is to grade the answer.
2. **Question text** – the full wording of the SAQ.
3. **Grading principles** – the rubric that distinguishes strong from weak answers, with short explanations.
4. **Gold answers** – instructor-graded feedback and grades for about ten answer chosen at random (about 9-19% of submissions; see Table 1).
5. **New answer** – the ungraded student response for the model to grade.

In all classes, instructors implemented our approach for AI-assisted grading using OpenAI’s GPT-4, the most capable LLM available during the study [22]. The LLM-generated feedback and numerical grade were extracted and entered into the course’s learning platform for students to review. For Gold Answers, instructors provided the grade and feedback directly. Although instructors had the freedom to adapt the approach, coordination across semesters minimized variation. The one exception was Class 4, which is excluded from discussion and analysis in the main text, as explained in S1 Appendix.

We chose GPT-4 over an open-source model, prioritizing ease of use and stronger performance over replicability and transparency [23]. Lower future replicability (when companies retire older models in the future) and less transparency (model parameters are not public) may be consequential for research and cumulation of scientific knowledge, but far less so when specific grades are rarely revisited weeks or months later. Ongoing review by instructors and students—who see and engage with the output—also reduces concerns about transparency. We revisit these concerns in the Conclusion.

We implemented the approach across four classes, collecting data from 271 students, 26 tests, and 3,080 graded SAQs. While student demographics varied slightly by class, we gathered information on age, in-state residency, first-generation status, anticipated work hours, and self-reported ACT/SAT percentiles (see S3 Appendix for question wording). As shown in Table 1, Class 1 had the most observations, younger students, and fewer average work hours. Class 2 skewed older with more work hours. Rates of in-state residency and ACT/SAT percentiles were similar across classes.

**Table 1:** (Sub)Sample summary statistics of classes.

	Pooled	Class 1	Class 2	Class 3	Class 5
# Participating students	271	90	38	52	91
% Enrolled participating	0.99	0.99	1.00	0.96	1.00
# Tests	26	7	4	10	5
# SAQs	48	17	14	10	7
# Gradings	3,080	1,510	499	458	613
Human graded (proportion)	0.12	0.11	0.19	0.14	0.09
Teaching assistant	-	Yes	No	No	Yes
Age (mean)	19.64	19.08	21.48	19.00	20.00
First generation (share)	0.15	0.16	0.19	0.08	0.15
ACT/SAT percentile (mean)	0.80	0.80	0.82	0.81	0.80
Work hours/ week (mean)	6.74	4.43	12.39	4.43	9.06
Study in home state (share)	0.58	0.57	0.59	0.63	0.54

Table notes: Summary statistics for all classes.

## Analysis

We evaluate our AI-assisted grading approach using three primary metrics, which we detail further below.

1. **Grade discrimination**: How well an SAQ’s score reflects each student’s overall mastery of the material.
2. **Regrade requests**: The frequency of students requesting human regrades of SAQs, which serves as a practical signal of grading errors that students deemed significant enough to contest.
3. **Perceived feedback helpfulness**: Students’ subjective evaluations of how helpful they found the written feedback.

Our main evaluations rely on pooled estimates across all classes. However, we report disaggregated results for key student subgroups: those scoring above and below the median ACT/SAT percentile, and first-generation college students. These subgroups are normatively and empirically relevant for understanding who benefits from AI tools. Given class-level heterogeneity and the overrepresentation of Class 1 (49% of the pooled sample), we also present results disaggregated by class.

To address minor missingness in demographic variables (0.5% for first-generation status; 0.8% age; 10.2% work hours; 13.7% ACT/SAT percentile), we implement multiple imputation with 20 completed datasets and average results across imputations [8,9]. All models include controls for whether students worked more than 20 hours per week, did not work, are first-generation, study in-state, are under age 20, and the logarithm of their ACT/SAT percentile. Standard errors are clustered at the student level. All data and replication code are available on the Open Science Foundation website: https://osf.io/7auxr/.

### Discrimination

We first examine how well each grading method—AI versus human—reflects a student’s broader mastery of material. This is a key metric for test evaluation, commonly reported as “discrimination” in learning platforms like Canvas or Blackboard. Since answers to SAQs were randomly assigned to human and AI grading, any differences in discrimination between AI and human graders reflects the grading method. Importantly, students’ other responses or scores did not directly inform the score for a given SAQ answer.

To test for differential discrimination, we examine how performance on other MCQs and SAQs, measured as the percentage of points earned, predicts the score for a specific SAQ. The outcome is the percentage of points awarded for that SAQ, with demographic controls included. Following the approach by Bansak (2021) [2], we estimate separate models for AI and human grading, then compare the coefficients on other MCQs/SAQs across these models. The difference in coefficients indicates how the grading method moderates the relationship between latent aptitude and SAQ scores. Full regression results are reported in S2 Table and S3 Table.

**Fig 1:**
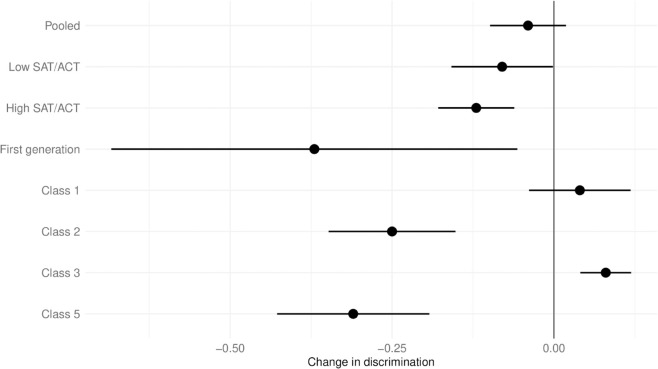
Predicting score by score on other questions (discrimination) by grading method. For each sample given on the y-axis, the x-axis shows how human grading differs from AI grading. Negative values mean that the AI approach translates scores on other test items more strongly into points than a human does. The dot gives the mean estimate, the line the 95% confidence interval.

Fig 1 shows the results for the difference in coefficients. The dot gives the mean estimate, the line the 95% confidence intervals. In the pooled sample, we find that AI discriminates more than a human grader on average. However, the difference is not significant (mean: −0.04, SE: 0.03) and the magnitude is miniscule.

The subset results reveal notable heterogeneity. In two classes, AI grading leads to a statistically significant decrease in discrimination; in one class, it results in a statistically significant increase. The magnitude of differences across sub-samples is generally modest. Even for the outlier result of Class 5, AI grading causes only a small effect: a one-standard deviation increase in performance on other test items (SD = 15.37 percentage points) to predict approximately 4.9 percentage points more on the SAQ (0.32×15.37), relative to human grading.

### Regrade requests

Per class policies, students could request a human regrade of any SAQ. This policy gave students an easy metaphorical fire alarm option [14]. We use these requests to evaluate the rate of severe grading errors.

**Fig 2:**
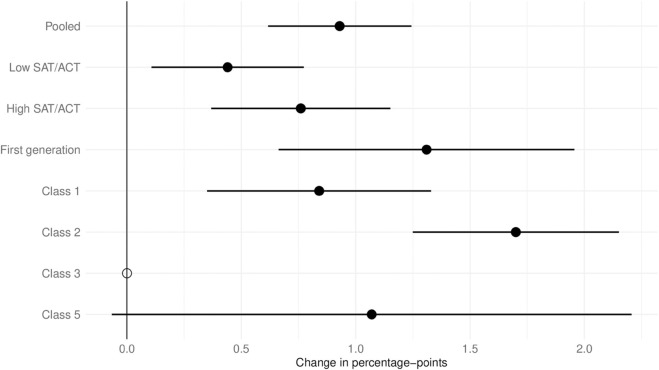
Regrade request by grading method. For the sample on the y-axis, the x-axis shows by how many percentage-points a human-graded SAQ draws a regrade request from a student compared to AI-graded questions. Positive values suggest that the AI-graded ones have fewer regrade requests. The empty circles indicate that in these classes, no regrade requests were made by students so that the coefficient is not estimable.

We estimate a linear probability model with an indicator variable for the grading method (1 for human grading; AI-grading as baseline) and with the same covariates as in previous analyses, clustering errors at the student level. Fig 2 shows the coefficients for the grading method indicator; the regressions are available in S1 Table. Overall, human-graded cases are more likely to result in regrade requests compared to AI-graded cases (mean: 0.93, SE: 0.16). However, the effect size is tiny. This result holds across almost all subsets. Notably, Class 3 had no regrade requests, making estimates unavailable.

Three points are important when interpreting the results. First, no students requested regrades for receiving too many points; thus, our analysis reflects concerns about under-grading. The latter, according to prior work [12], can harm future learning. However, unlike that study, we provided extensive personalized written feedback on students’ work, likely mitigating the effects of falsely positive grades.

Second, since grades could be lowered through human regrades, some students probably did not submit requests, doing so only when they believed a significant adverse error occurred. This policy likely discouraged frivolous or opportunistic regrade requests, meaning the regrade requests we analyze likely reflect serious concerns from students. Notably, the only impactful request across classes resulted from a human copy-paste error when transferring LLM output to the learning platform.

Third, the low overall regrade request rate (1.41% in the pooled sample) may reflect the university’s academic culture, where students rarely contest grades. Other institutions may see higher regrade rates depending on their academic culture.

### Retrospective subjective evaluation of feedback helpfulness

On each test, students were asked how helpful they found the feedback on their previous test answers, ranging from “very unhelpful” to “very helpful,” with options to skip or indicate they did not take the last test. Although the question does not measure learning, it captures perceived helpfulness, which may encourage introspection and engagement. For analysis, we create binary outcome variables for any “helpful” and any “unhelpful” evaluations.

The sample is limited to students who took both the current and previous tests, using the test as the unit of analysis. In the pooled sample, 10.0% gave an “unhelpful” rating, and 60.5% gave a “helpful” one, or 12.4% and 74.7%, respectively, among those giving substantive responses (ie. responses that expressed helpfulness or unhelpfulness to any extent). The key explanatory variable is whether any SAQ on the last test is human-graded (19.5% of observations). Tests with no human feedback make up 80.5%, and those with all human grading are 6.8%; on average, 12.1% of questions are human graded. This binary indicator is thus coarser and noisier than the earlier SAQ-specific one. We apply the same linear regression approach with clustered standard errors and estimate coefficients separately for each outcome. Regression tables appear in S4 Table and S5 Table.

**Fig 3:**
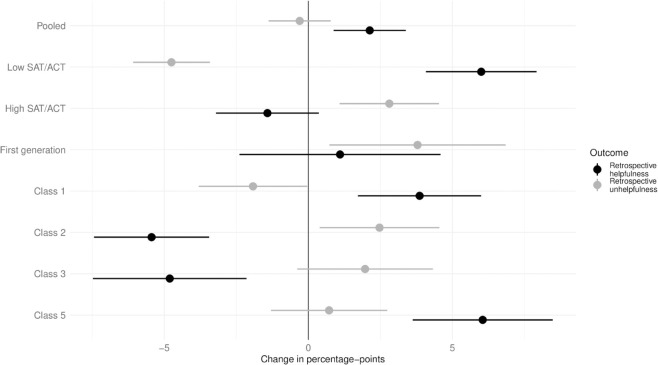
Retrospective evaluation of feedback helpfulness. The black dots/lines gives the coefficient estimate and confidence interval when regressing an indicator for whether the feedback was helpful in any way on an indicator for whether any question was graded by a human. The gray analogues show the results for when outcome is any retrospective unhelpful evaluation.

Fig 3 presents the results. Human grading has no statistically significant effect on unhelpful evaluations (mean: -0.30, SE: 0.50), but increases the probability of a helpful evaluation in a statistically significant but modest way (mean: 2.13, SE: 0.64). Among lower-ACT/SAT percentile students, helpfulness increases (mean: 6.00, SE: 0.98) and unhelpfulness decreases (mean: −4.75, SE: 0.68), though both effects remain small. Results for higher percentile students are similar in direction but even smaller in size.

Results vary by class. In Classes 1 and 5, human feedback is more likely to be viewed positively in retrospect, while in Classes 2 and 3, the opposite is true. Despite the negative evaluations in Classes 2 and 3, it is possible students recognize instructor-written feedback and respond favorably to the human touch. However, since regrade requests are more common for human-graded SAQs—implying students would be asking the grader to reassess their own work—it is unclear whether students actually know who performed the grading. This contrasts with studies showing LLM-generated feedback is perceived as more empathetic [10,31]. Still, all effects are small, generally under five percentage points. An additional (non-pre-registered) analysis using an alternative indicator—comparing fully human-graded tests to all others—produces similar results: mostly small and statistically insignificant effects (see S6 Table and S7 Table).

## Conclusion

Our RCT across undergraduate political science classes in 2023/2024 suggests that detailed grading typical of small classes can scale to larger ones using generative AI tools like ChatGPT, Gemini, or Claude. AI–generated feedback and grades generally mirror those of human instructors, with average differences of tiny magnitudes. Therefore, affords large productivity gains for instructors grading SAQs.

Instructors may remain wary of LLM grading, however, citing opacity, hallucinations, and ideological tilt [27]. A further worry is that built-in political bias could penalize students who offer heterodox views. Yet human grading is hardly immune to unfairness: rubrics are often implicit, judgments drift with fatigue, and personal leanings—or impressions of a student—can seep into grades. Issues hidden in a model mirror those hidden in a tired grader’s mind. In contrast with humans, an LLM applies the same, visible approach every time. A prompt can be re-run on the identical answer (without knowledge of its author) to reproduce the same result, insulated from lapses in attention, moment-to-moment mood, or favoritism. Our study was not designed to examine ideological skew, instructor favoritism, and lack of grading fatigue, but we endorse future research doing so.

All in all, we see this study as a starting point for further research on using AI tools augment instructors’ productivity grading SAQs. Prompt templates need only a handful of instructor-graded examples; once built, they scale to hundreds of SAQs at negligible cost. Follow-p work can explore ensembles of LLMs, use richer prompts [28], and extend the approach from SAQs to full-length essays. Ongoing improvements in model capability [15] and falling usage costs will let instructors iterate quickly and thus to improve the alignment AI grading with their pedagogical goals.

## Supporting information

S1 Appendix Deviations from pre-analysis plan. (TIF)

S2 Appendix Student survey and informed consent. (TIF)

S3 Appendix Overview of demographic questions and variables. (TIF)

S4 Appendix Illustration of AI-grading approach. (TIF)

S1 Table Coefficient estimates for regrade requests models. The first number gives the mean estimate, the number in parentheses the standard error. The column gives the (sub)sample used. Models for Class 3 and 4 were not estimated as there was no variation for the outcome.
(TIF)

S2 Table Coefficient estimates for discrimination (human-graded) models. The first number gives the mean estimate, the number in parentheses the standard error. The column gives the (sub)sample used.
(TIF)

S3 Table Coefficient estimates for discrimination (AI-graded) models. The first number gives the mean estimate, the number in parentheses the standard error. The column gives the (sub)sample used.
(TIF)

S4 Table Coefficient estimates for subjective retrospective helpfulness models. The first number gives the mean estimate, the number in parentheses the standard error. The column gives the (sub)sample used.
(TIF)

S5 Table Coefficient estimates for subjective retrospective unhelpfulness models. The first number gives the mean estimate, the number in parentheses the standard error. The column gives the (sub)sample used.
(TIF)

S6 Table Coefficient estimates for subjective retrospective helpfulness models when comparing all-human grading to not-all human grading. The first number gives the mean estimate, the number in parentheses the standard error. The column gives the (sub)sample used.
(TIF)

S7 Table Coefficient estimates for subjective retrospective unhelpfulness models when comparing all-human grading to not-all human grading. The first number gives the mean estimate, the number in parentheses the standard error. The column gives the (sub)sample used.
(TIF)
