# Supplementary figures and images for: AI-assisted grading and personalized feedback in large political science classes: Results from randomized controlled trials
Source: PLoS One. 2025 Aug 19;20(8):e0328041. doi: 10.1371/journal.pone.0328041 (PMC12364334; doi:10.1371/journal.pone.0328041)

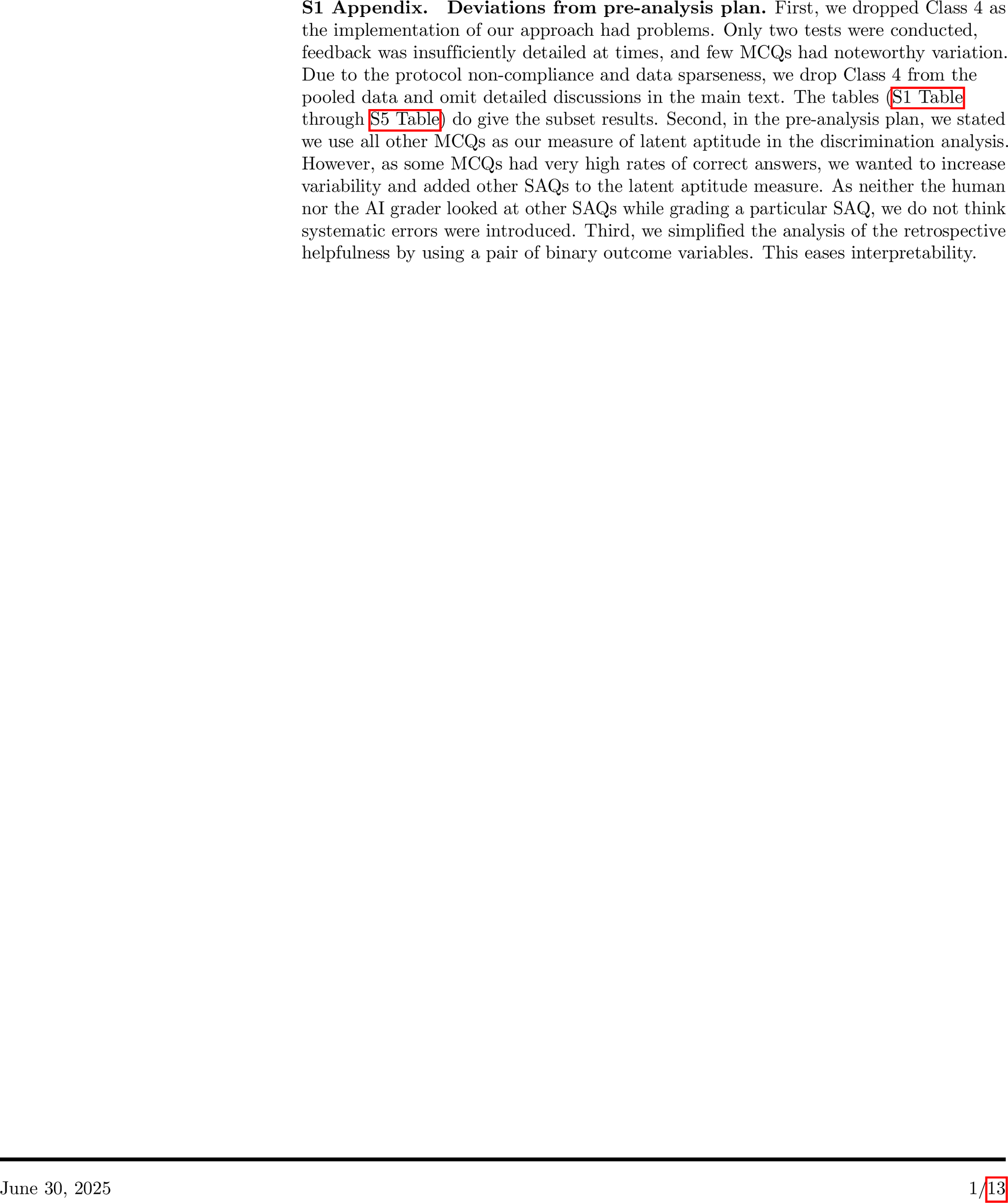

Supplement: S1 Appendix — (TIF) [file pone.0328041.s001.tif]

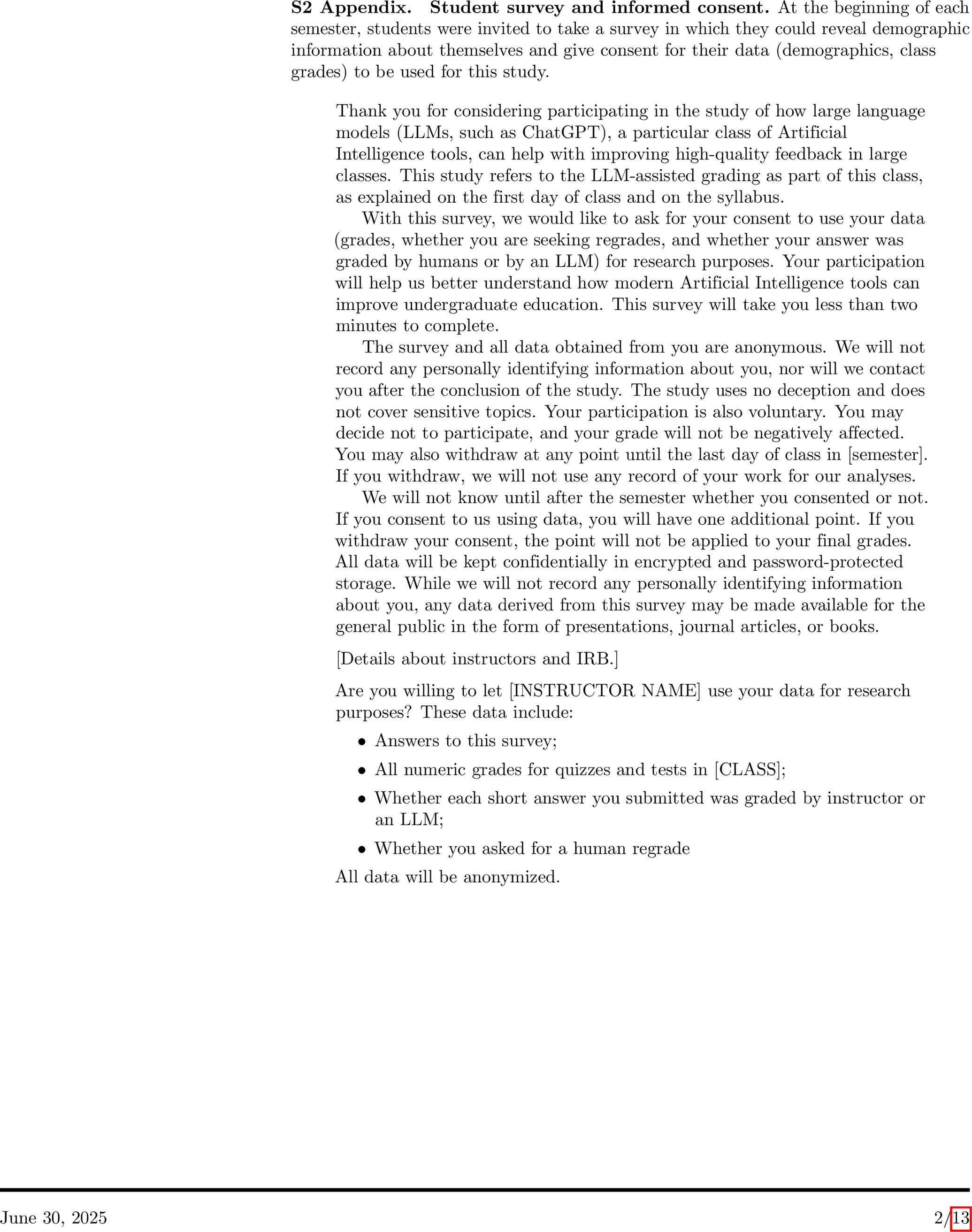

Supplement: S2 Appendix — (TIF) [file pone.0328041.s002.tif]

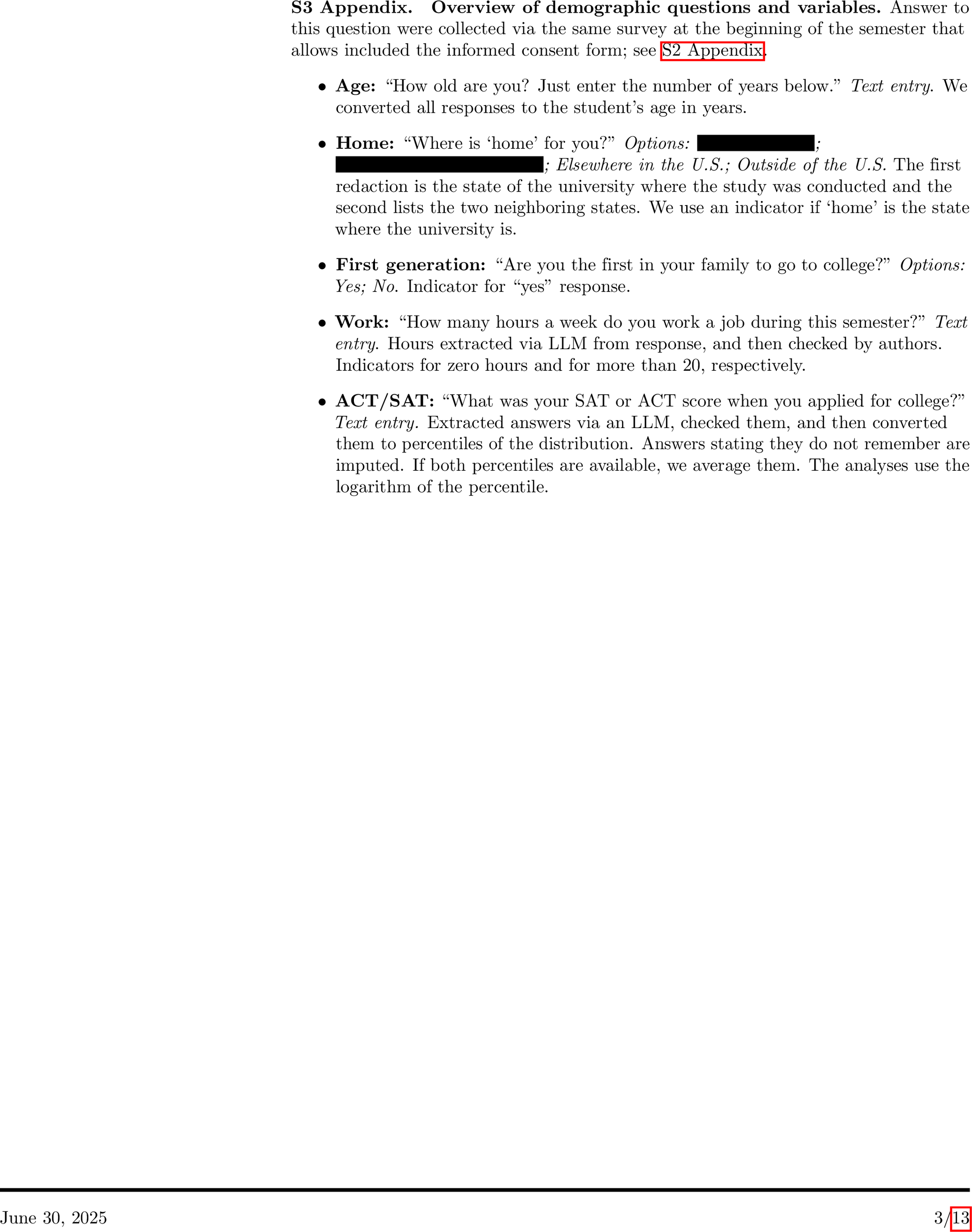

Supplement: S3 Appendix — (TIF) [file pone.0328041.s003.tif]

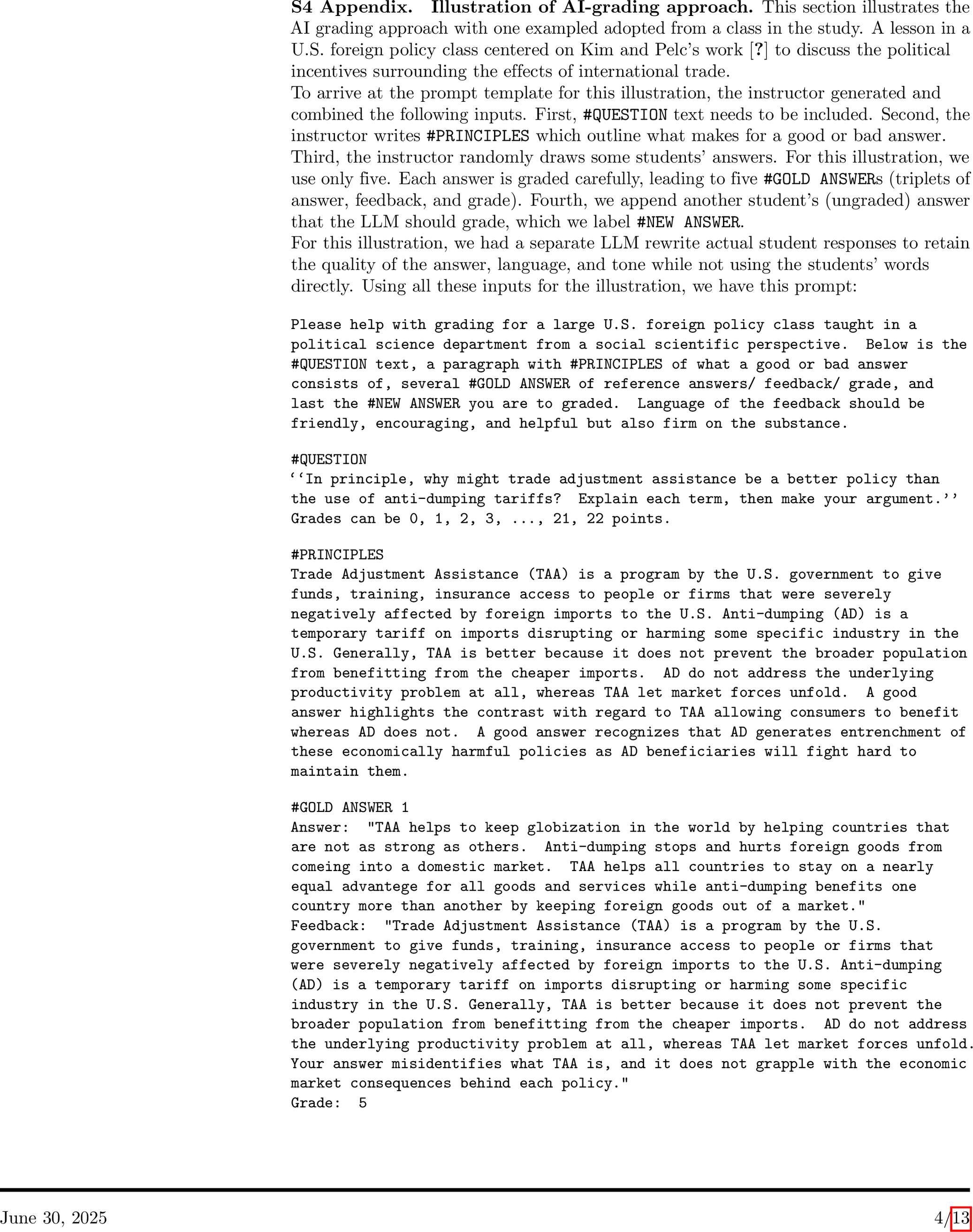

Supplement: S4 Appendix — (TIF) [file pone.0328041.s004.tif]

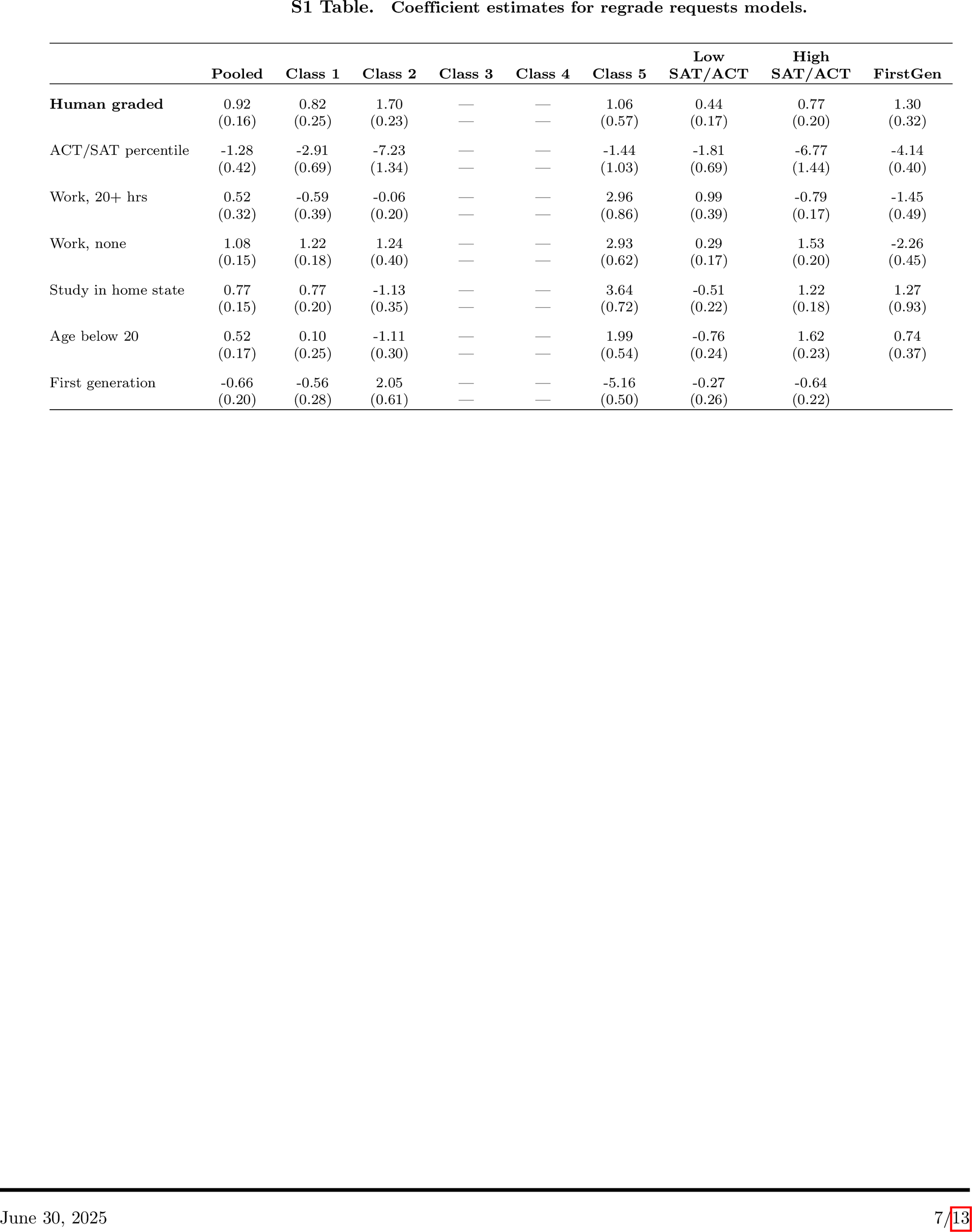

Supplement: S1 Table — The first number gives the mean estimate, the number in parentheses the standard error. The column gives the (sub)sample used. Models for Class 3 and 4 were not estimated as there was no variation for the outcome. (TIF) [file pone.0328041.s005.tif]

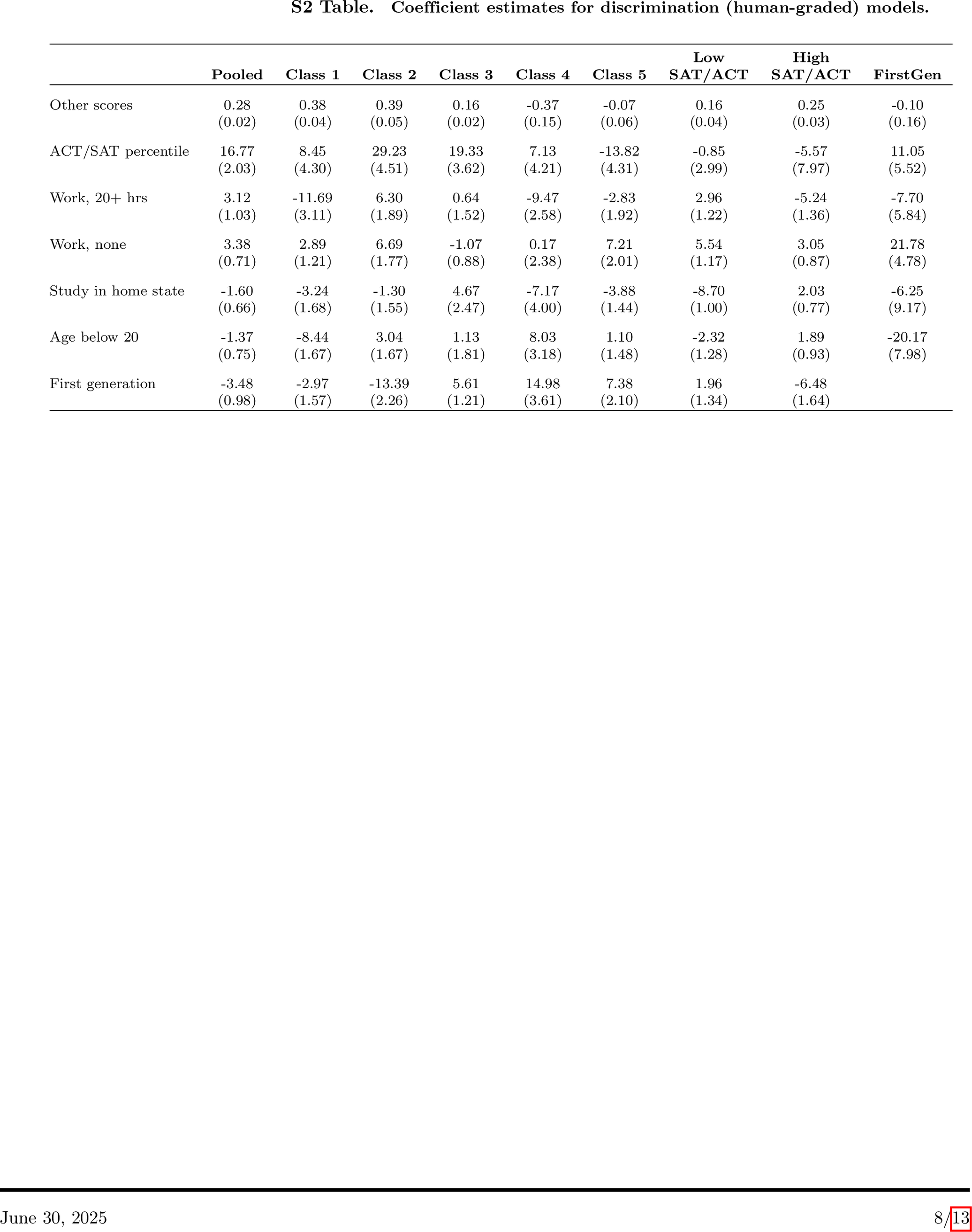

Supplement: S2 Table — The first number gives the mean estimate, the number in parentheses the standard error. The column gives the (sub)sample used. (TIF) [file pone.0328041.s006.tif]

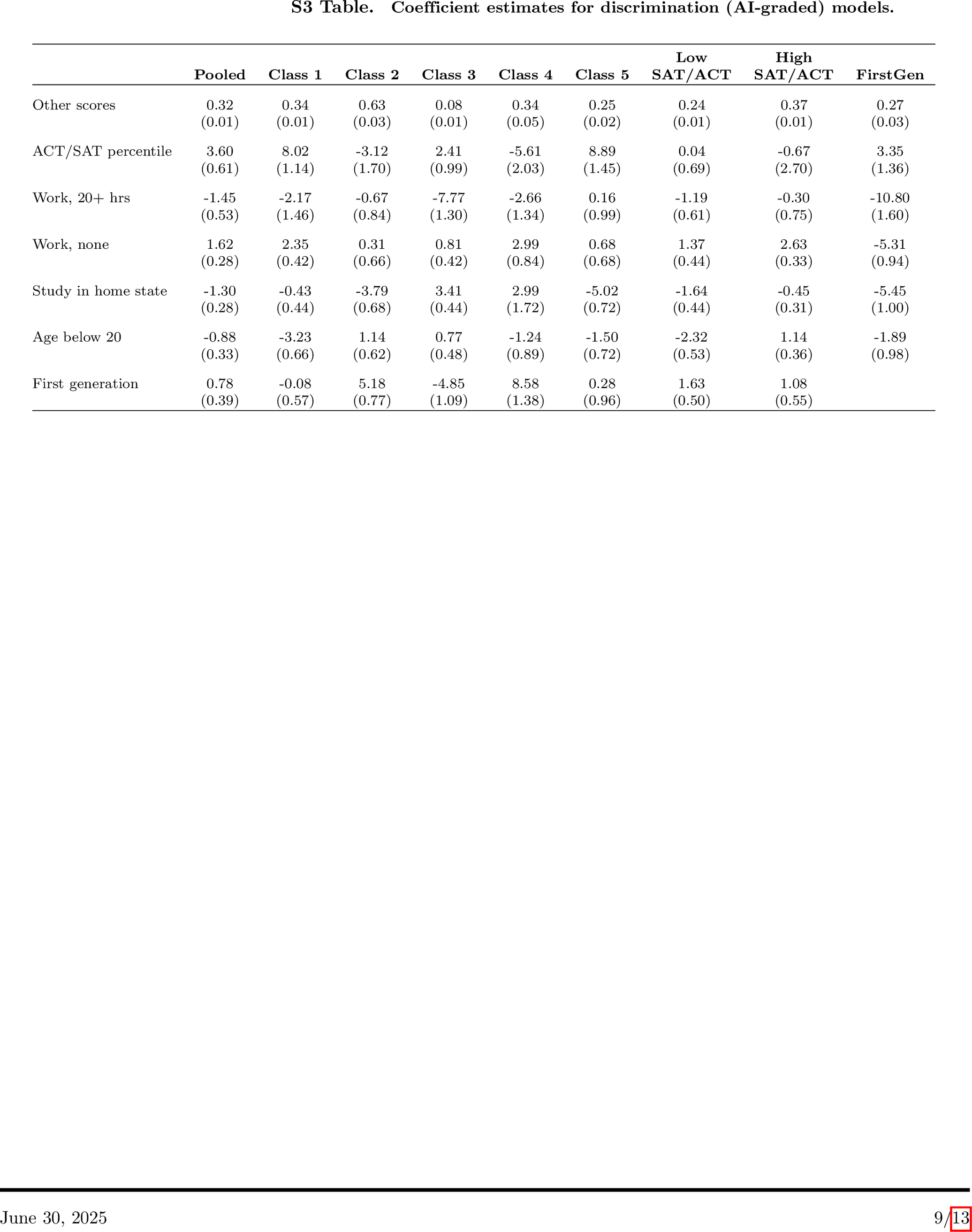

Supplement: S3 Table — The first number gives the mean estimate, the number in parentheses the standard error. The column gives the (sub)sample used. (TIF) [file pone.0328041.s007.tif]

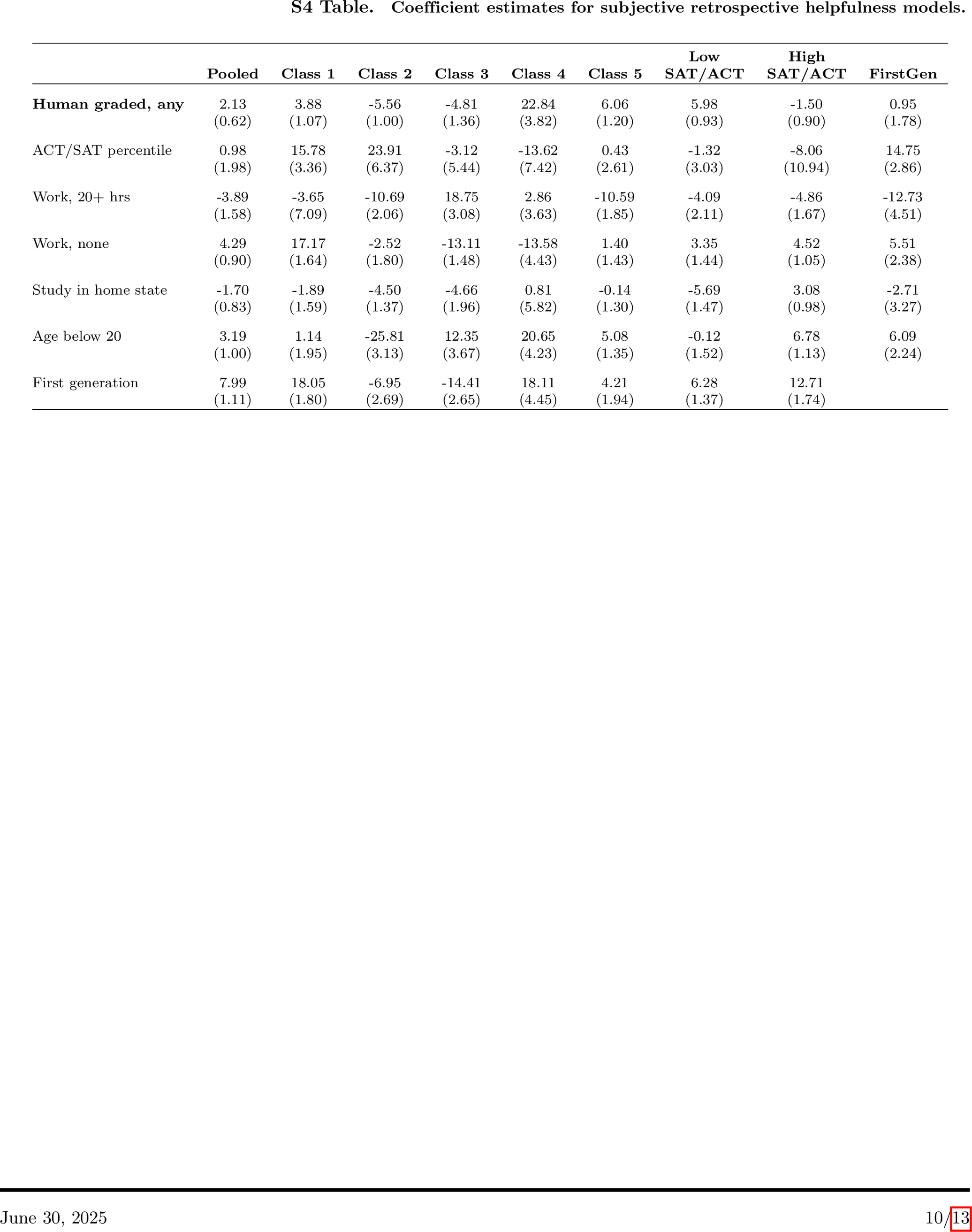

Supplement: S4 Table — The first number gives the mean estimate, the number in parentheses the standard error. The column gives the (sub)sample used. (TIF) [file pone.0328041.s008.tif]

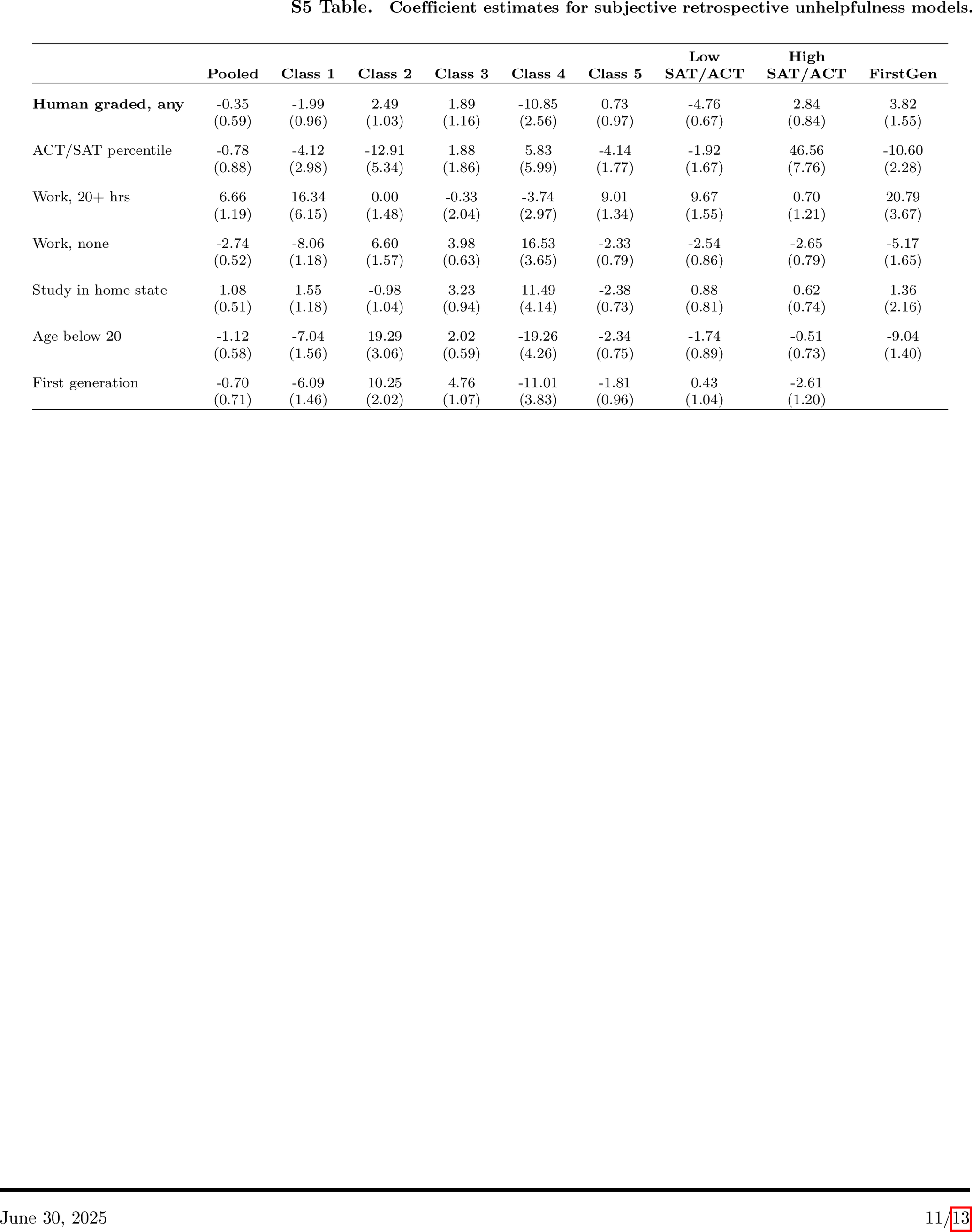

Supplement: S5 Table — The first number gives the mean estimate, the number in parentheses the standard error. The column gives the (sub)sample used. (TIF) [file pone.0328041.s009.tif]

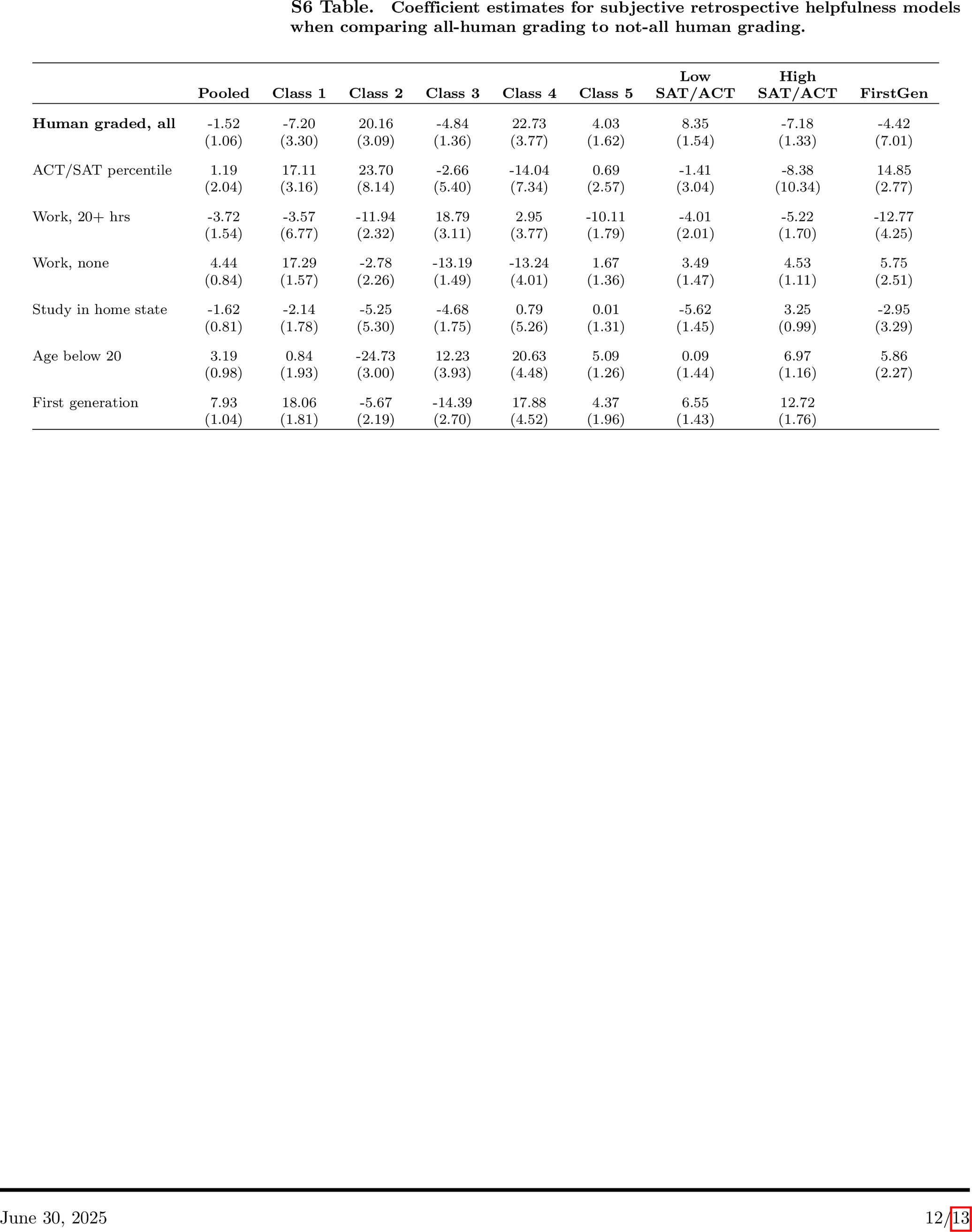

Supplement: S6 Table — The first number gives the mean estimate, the number in parentheses the standard error. The column gives the (sub)sample used. (TIF) [file pone.0328041.s010.tif]

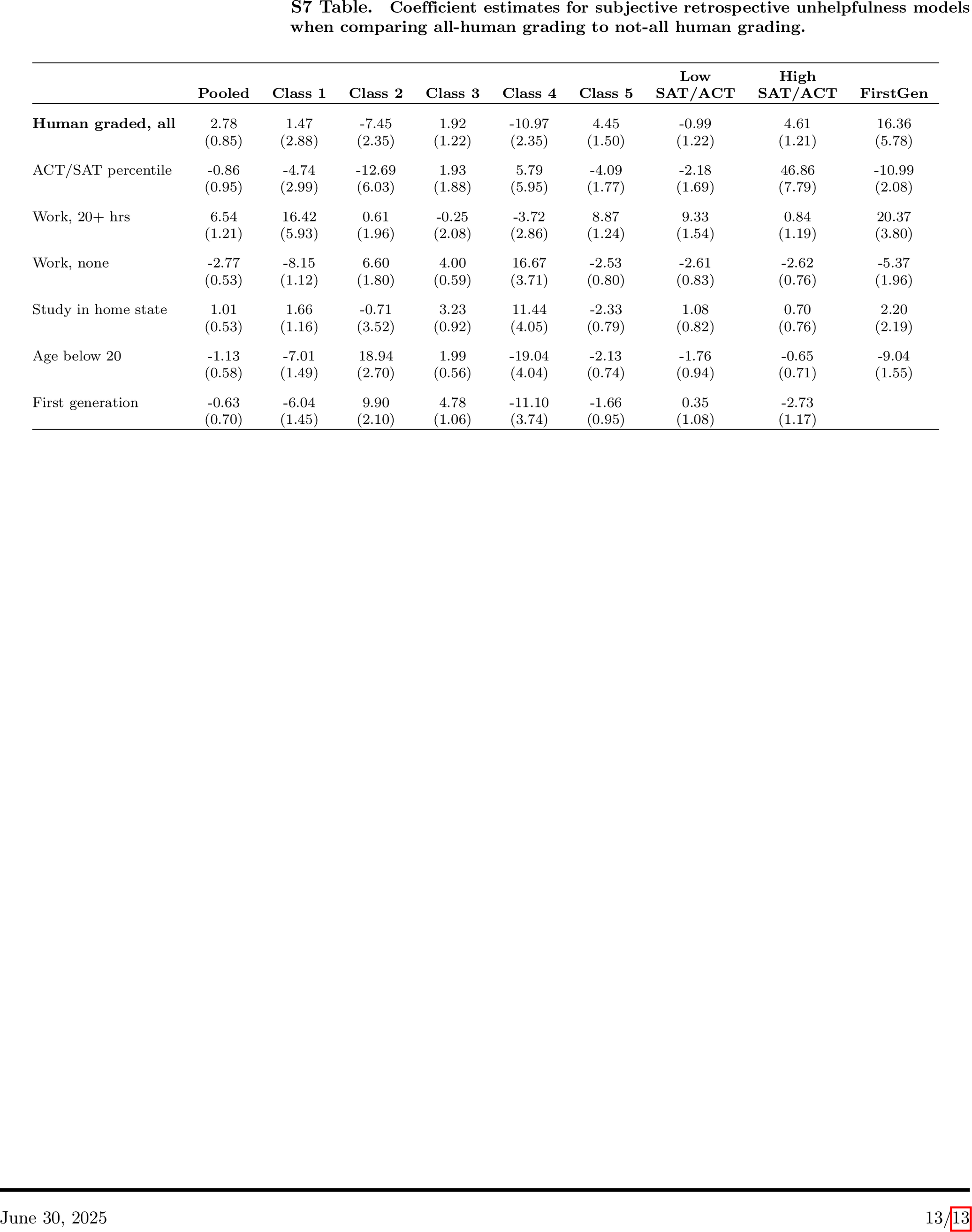

Supplement: S7 Table — The first number gives the mean estimate, the number in parentheses the standard error. The column gives the (sub)sample used. (TIF) [file pone.0328041.s011.tif]
